# Supplementary material for: A feasibility study demonstrating that independence, quality of life, and adaptive behavioral skills can improve in children with Down syndrome after using assistive technology
Source: PLoS One. 2023 May 24;18(5):e0284738. doi: 10.1371/journal.pone.0284738 (PMC10208506; doi:10.1371/journal.pone.0284738)
Supplement: S1 Protocol — (DOCX) [file pone.0284738.s001.docx]

Umbrella Study to Evaluate the Feasibility of Collecting User Interaction Data in Participants Using MapHabit Software

Protocol Number: MH001

National Clinical Trial (NCT) Identified Number: NCT05343468

Principal Investigator: Stuart Zola, PhD

Funded by: National Institute of Health / MapHabit, Inc.

Version Number: v.0.2

1 AUG 2020

**Summary of Changes from Previous Version:**

| **Affected Section(s)** | **Summary of Revisions Made** | **Rationale** |
| --- | --- | --- |
| 1, 2, 4, 5, 6, 9, 10 and 16 | Protocol Summary has been modified to include a population of children ages 5-17 with Down syndrome. Furthermore, a set of assessments and questionnaires have been added in order to assess each of the population groups for sub-studies. | Initial Version included only an older adult population of participants with Alzheimer’s Disease and other related dementias or cognitive impairments. Additionally, the initial version of this document did not include the assessments to be potentially implemented in sub-studies for either population. |
|  |  |  |
|  |  |  |
|  |  |  |
|  |  |  |
|  |  |  |
|  |  |  |

#

#

[1. **Protocol Summary** 4](#_Toc42794632)

[1.1. Synopsis 4](#_Toc42794633)

[1.2. Schedule of Activities 7](#_Toc42794634)

[2. **Introduction** 7](#_Toc42794635)

[2.1. Study Background and Rationale 7](#_Toc42794636)

[2.2. Description of the MapHabit Software used in Sub-studies 8](#_Toc42794637)

[3. **Objectives and Outcomes** 9](#_Toc42794638)

[4. **Study Design** 9](#_Toc42794639)

[4.1. Treatment Assignment 10](#_Toc42794640)

[5. **Study Population** 11](#_Toc42794641)

[5.1. Inclusion Criteria 11](#_Toc42794642)

[5.2. Exclusion Criteria 12](#_Toc42794643)

[5.3. Withdrawal Criteria 12](#_Toc42794644)

[5.4. Screen Failures 12](#_Toc42794645)

[6. **Study Methods** 12](#_Toc42794646)

[6.1. Participants Joining the Umbrella Study from Sub-Study 12](#_Toc42794647)

[6.2. Pre-recruitment 12](#_Toc42794648)

[6.3. Enrollment: Day 0 12](#_Toc42794649)

[6.4. Data Collection Period 13](#_Toc42794650)

[6.5. Follow Up Assessments 13](#_Toc42794651)

[7. **Statistical Considerations** 13](#_Toc42794652)

[7.1. Sample Size Determination 14](#_Toc42794653)

[8. **Subject Accountability** 14](#_Toc42794654)

[8.1. Withdrawal 14](#_Toc42794655)

[9. **Safety** 14](#_Toc42794656)

[9.1. Risk/Benefit Assessment 14](#_Toc42794657)

[9.1.1. Known Potential Risks 14](#_Toc42794658)

[9.1.2. Known Potential Benefits 14](#_Toc42794659)

[9.1.3. Assessment of Potential Risks and Benefits 15](#_Toc42794660)

[9.2. Unanticipated Problems 15](#_Toc42794661)

[9.2.1. Definition of Unanticipated Problems (UP) 15](#_Toc42794662)

[9.2.2. Unanticipated Problem Reporting 15](#_Toc42794663)

[9.2.3. Serious Adverse Event (SAE) Reporting 16](#_Toc42794664)

[9.2.4. Adverse Device Event Reporting 16](#_Toc42794665)

[9.3. Death Reporting 17](#_Toc42794666)

[10. **Informed Consent** 17](#_Toc42794667)

[10.1. Assessment of Capacity for Informed Consent 18](#_Toc42794668)

[10.2. Process of Obtaining Informed Consent Through Surrogacy 18](#_Toc42794669)

[11. **Protocol Deviations** 18](#_Toc42794670)

[12. **Study Discontinuation and Closure** 18](#_Toc42794671)

[13. **Data Management** 19](#_Toc42794672)

[13.1. Data analysis plan 19](#_Toc42794673)

[13.2. Quality control 19](#_Toc42794674)

[13.3. Data handling 19](#_Toc42794675)

[13.4. Data Collection 20](#_Toc42794676)

[13.5. Study Records Retention 20](#_Toc42794677)

[14. **Abbreviations** 20](#_Toc42794678)

[15. **Statement of Compliance** 21](#_Toc42794679)

[16. **Potential Sub-Study Surveys & Questionnaires** 23](#_Toc42794680)

[17. **Assent Form** 77](#_Toc42794695)

[18. **References** 81](#_Toc42794697)

#

#

# Protocol Summary

## Synopsis

**Title**

Umbrella Study to Evaluate the Feasibility of Collecting User Interaction Data in Participants Using MapHabit Software

**Study Description**

This is a multi-center, prospective, observational, sample collection/methods development study, with continuous user interaction data collection.

**Objectives**

1. Primary Objective:

- Demonstrate the feasibility of collecting user interaction data in participants using MapHabit software

1. Exploratory Objective:

- For AD/RD: Develop measures to qualify and quantify disease progression and severity based on the collected user interaction data. Participants will be expected to utilize the MapHabit application either independently or with the assistance of a caregiver/LAR depending on the capacity of the participant.
- For DS: Develop measures to qualify and quantify independence and improvements in quality of life. Participants will be expected to utilize the MapHabit application under the supervision of the parent or legal guardian.

**Endpoints**

1. Primary Endpoint:

- Availability of user interaction data
- A set of sub-study specific questionnaires at the start and end of the intervention

1. Exploratory Endpoint:

- Mathematical model to establish a relationship between: behaviors while operating a mobile device as measured by user interaction data collected during this study, and disease severity and progression as measured in subsequent sub-study(ies) conducted by MapHabit Inc.

**Inclusion, Exclusion and Withdrawal Criteria:**

1. Inclusion Criteria:

- **For Adult population with AD, ADRD, TBI, MCI, or other form of cognitive impairment:** Female or male subjects must be over the age of 18. A legally authorized representative (LAR) must sign on behalf of the subject if the subject does not have the capacity to consent
- **For Down Syndrome Population**: Females and males 5-17 years of age. Parents or Legal guardians must sign for subjects under the age of 18
- A clinical diagnosis of Alzheimer’s Disease (AD), Alzheimer’s Disease Related Dementia (ADRD), Mild Cognitive Impairment (MCI), Traumatic Brain Injury (TBI), other forms of cognitive impairment OR a diagnosis of Down Syndrome (DS) OR a caregiver of an individual within the two populations above
- Candidate participants over the age of 18 with clinical diagnosis of AD/ADRD, MCI, TBI, or other form of cognitive impairment OR Down Sydnrome are screened for capacity to provide Informed Consent (see Section 10)
- Informed Consent obtained from candidate participant or legally authorized representative (LAR), parent, or legal guardian as appropriate

1. Exclusion Criteria:

- Candidate participants with capacity and unwilling to provide Informed Consent for this study, or candidate participants without capacity and unable to provide Informed Consent through surrogacy, are excluded from enrollment

1. Withdrawal Criteria:

- Withdrawal of Informed Consent
- For caregivers of individuals with AD/ADRD, MCI, TBI, or other form of cognitive impairment: dissolution of a suitable patient-caregiver relationship

**Study Population:**

The maximum number of participants enrolled in the study is 1000. The participants will be using the MapHabit software in scope of MapHabit Inc. sub-studies.

Cohort 1: Must be 18 years of age or older at the time of consent. This study will enroll individuals with cognitive impairments (n=<300) and the caregivers of individuals with cognitive impairment (n ~ 250). Caregivers may be associated with more than one individual. Enrolled individuals with cognitive impairment must have had a previously established clinical diagnosis of Alzheimer’s Disease (AD), Alzheimer’s Disease Related Dementia (ADRD), Traumatic Brain Injury (TBI), Mild Cognitive Impairment (MCI) or other forms of cognitive impairment.

Cohort 2 (Amended Population): Must be between the ages of 5-17 years old at the time of the study. This study will enroll individuals n =< 250 and the primary caregiver/parent of individuals with Down Syndrome (DS) (n ~ 250). Enrolled individuals with DS must have a previously established clinical diagnosis of DS. A parent or legal guardian must consent for subjects under the age of 18 and supervise the child’s use of the system.

**Recruitment**

Study participants will be recruited from the general public and within the MapHabit software. Recruitment material that is provided in-person, digitally, or through the MapHabit software will include:

- Purpose of the study
- Expectation of the participants
- Time commitment for participating
- Location where research is taking place

No compensation, either monetary or discount of the MapHabit software, is offered for participation in this study. Recruitment material presented within the MapHabit software will have design elements (e.g. font size, color, graphics) consistent with the remainder of the software interface. Reimbursement will be provided for expenses, including transportation to initial or exit interview, that may be incurred related to this study. The MapHabit System will be offered to participants during this study at no cost to the participants or families of the participants during the course of this study.

**Description of Study Intervention:**

This umbrella study does not introduce any intervention other than the utilization of the MapHabit System during the specified period of the sub-study. Furthermore, subsequent MapHabit Inc. sub-studies may measure cognitive and health outcomes of all study participants. The user interaction data collected in this umbrella study, and clinical outcomes data collected in subsequent sub-studies can be analyzed in combination to generate a mathematical model demonstrating the relationship between these two types of measurements. It is hypothesized that such a mathematical model may be used to forecast future changes in cognition and health based on behaviors while operating a mobile device. This forecast may be used by clinicians to guide changes in medical and behavioral therapy, hospital systems and insurers to identify at-risk and high utilization patients, and individuals with cognitive impairment and family members better prepare emotionally and financially for the future.

**Participant Duration:**

This umbrella study protocol does not require a minimum or maximum duration of study participation. The duration of participation in a research study will be defined by subsequent MapHabit, Inc. sub-study(ies) in which the subjects or their LAR, parent/legal guardian (where applicable) will participate in an exit interview at the end of the study. The study will be completed once the maximum number of participants complete the study. Participants in the AD/RD sub-study population will be expected to use the MapHabit System daily for 6 months. Participants in the DS sub-study population will be expected to use the MapHabit System for 3-6 months. Participants may use the MapHabit System for up to 2 years, in which data will be passively collected with no additional expectations of the users.

## Schedule of Activities

| **Study Activity** | **Enrollment**  **(Day 0)** | **Continuously** | **End of Participation** |
| --- | --- | --- | --- |
| Inclusion/Exclusion screening | X |  |  |
| Participant completes enrollment to this study within the MapHabit software | X |  |  |
| Participant is evaluated for ability to provide Informed Consent | X |  |  |
| Informed Consent is provided by participant or consenting legally authorized representative | X |  |  |
| Assignment of unique participant number | X |  |  |
| Initial Baseline Interview with sub-study appropriate questionnaire or survey | X |  |  |
| Passive data collection |  | X |  |
| Evaluation of Withdrawal Criteria |  | X |  |
| Study completion |  |  | X |
| Exit Interview with sub-study appropriate questionnaire or surveys throughout study |  |  | X |
| Post intervention survey as appropriate for sub-study |  |  | X |

# Introduction

## Study Background and Rationale

As Alzheimer’s disease (AD) progresses, an increasing rate of atrophy is seen in the hippocampus [1] while the neostriatum is preserved [2]. The neostriatum is responsible for the development and retention of procedural memory, such as routines and habits.

Down Syndrome (DS) occurs in one in 700 live births. In addition to intellectual disability, people with DS are very likely to eventually develop AD. 70% of people with DS develop the neuro-pathophysiology associated with AD by their 40s, and current research suggests that there could be common pathogenic mechanisms [6]. Importantly, such mechanisms primarily manifest as learning disabilities in children with DS, involving mild to moderate cognitive impairment [7]. Evidence suggest early intervention that address cognitive, language, and social-emotional needs of children with DS can promote child development. Specifically, engagement in strongly responsive interactions with daily routines induce the child’s use of various pivotal developmental behaviors, such as attention, initiation, and persistence [8].

The MapHabit software was developed as an assistive technology for individuals living with AD, Alzheimer’s disease related dementias (ADRD), and their caregivers. This technology utilizes aspects of visual mapping to enhance the ability of memory impaired individuals to live independently. Visual mapping is a learning technique to diagram relationships between ideas and memories. Despite remarkable success across a wide range of disciplines, there is nothing to deliver the capabilities of visual mapping for individuals living with cognitive impairment. Currently commercial visual mapping software is too complicated for users with memory, cognitive, and physical impairments and are not easily adaptable. The MapHabit software combines features of mind mapping with a dynamically responsive user interface to assist individuals, caretakers, and clinicians.

Here we propose an umbrella trial to evaluate the feasibility of collecting information about user interactivity with the MapHabit software interface. This data has the potential to be used for further development of the MapHabit software and developing measures of disease severity, progression, and independence.

## Description of the MapHabit Software used in Sub-studies

By engaging with the MapHabit software, a user can develop and utilize visual maps to show schedules and activities of daily living (ADL). Visual mapping is a technique to enhance learning and memory that has been studied previously in cognitive intact populations [3][4]. It is hypothesized that the use of the visual maps will enhance the ability of patients with cognitive impairment to improve the quality of their life (QoL). The visual maps are personalized for each individual, created specifically for activities requiring additional assistance, and serve as a constant and available reminder of important information.

After repeated use of the MapHabit software, individuals with impaired memory can develop a habit of routinely using visual maps to independently manage their daily activities. Doing so alleviates dependence of individuals with cognitive impairment from their caregivers. Benefits of the MapHabit software include, but are not limited to, reduced levels of stress, agitation, confusion, frustration, and anger. Individuals become less reliant on caregivers and family members, achieving more autonomy in daily living. Stress and burden experienced by caregivers and family members are significantly reduced, improving QoL for all parties involved.

# Objectives and Outcomes

| **OBJECTIVES** | **OUTCOMES** | **JUSTIFICATION FOR OUTCOMES** |
| --- | --- | --- |
| **Primary Objective** |  |  |
| Demonstrate the feasibility of collecting user interaction data in participants using MapHabit software | Availability of user interaction data | We want to demonstrate that the data collection is technically feasible, and the structure of the collected data is suitable for analysis and measure development |
| **Exploratory Objective** |  |  |
| Develop measures of disease severity, progression, independence and quality of life with AD/RD participants based on the collected user interaction data. For DS participants, this study also hopes to develop measures for independence and quality of life based on the collected user interaction data | Mathematical model to establish a relationship between behaviors while operating a mobile device (as measured by user interaction data) and clinical disease severity and progression (as measured in subsequent sub-studies) | We hypothesize that a subject’s behavior within the software is affected by clinical severity and progression of disease and/or may influence the subject’s independence, quality of life, or other outcomes as measured by the sub-study specific questionnaires or surveys. |

# Study Design

Study Design for the ADRD Population:

This is a multi-center, prospective, observational, sample collection/methods development study, with continuous user interaction data collection. This study will enroll two groups of participants:

1. Individuals (n =<300) with a diagnosis of AD, ADRD, TBI, MCI, DS or other form of cognitive impairment. Individuals will participate in sub-study relevant surveys and questionnaires if they have the capacity to do so throughout the study period.
2. Caregivers OR Legally Authorized Representatives (n ~ 250) of enrolled individuals. One caregiver may be assigned to several individuals. Caregivers will participate in sub-study surveys if the individual does not have the capacity to do so.

Study Design for the DS Population:

This is a multi-center, prospective, observational, sample collection/methods development study, with continuous user interaction data collection. This study will enroll two groups of participants:

1. Individuals (n =< 250) between the ages of 5-17 with a diagnosis DS
2. Parents/Legal Guardians (n ~ 250) of the enrolled individuals. Parents must complete surveys and questionnaires on the behalf of the individual throughout the study period.

Study participants will engage with the MapHabit software through a mobile device (e.g. smart phone or tablet) equipped with MapHabit software. This study will collect measures of participant engagement within the MapHabit software via Google Analytics for Firebase platform. The collected data is then transferred to a cloud computing platform for processing and further analysis. Data originating from Google Firebase will be encrypted in transit using TLS/SSL and encrypted at rest using the AES-256 encryption algorithm. The following data types will be collected:

1. Dwell time: the duration of time that an element of the software interface is displayed to the participant without any further interaction. This can be further segmented to:
   1. Screen dwell time
   2. Prompt dwell time
2. User input: actions (e.g. tapping, swiping, typing) with all interactive elements (e.g. buttons, dialog box) within the MapHabit software will be recorded with an element identifier and time of interaction
3. Application logs, such as error reports, unexpected shutdowns, etc.

Participants are considered enrolled in this study after providing Informed Consent. Informed Consent may be obtained in writing or electronically.

The study will consist of three parts:

1. Enrollment in study (day 0)
2. Continuous data collection
3. End of participation

This umbrella study protocol does not require a minimum or maximum duration of study participation. The duration of participation in a research study will be defined by subsequent MapHabit, Inc. sub-study(ies) for participants with ADRD and separately for children with DS and their parents or legal guardians. The study will be completed once the maximum number of participants complete the study. The MapHabit System is easy to setup upon beginning the study and all participants will undergo an initial onboarding training virtually or in-person.

## Treatment Assignment

Any participant meeting all inclusion criteria is eligible for enrollment in this study. Participants are considered enrolled in this study after providing Informed Consent. Informed Consent may be obtained in writing or electronically.

# Study Population

The maximum number of participants enrolled in study this 1000. Participants in the first study population cohort are individuals with clinical diagnosis of AD, ADRD, TBI, MCI, or other form of cognitive impairment; and/or their respective caregivers; who are using the MapHabit software in scope of MapHabit sub-studies. All participants are at least 18 years of age on day of consent. No further demographic information regarding sex, gender, race, and location, income, marital status, or living situation is defined at this time. The study will enroll individuals with cognitive impairment (n = 250) and caregivers of individuals with cognitive impairment (n ~ 250). All individuals with cognitive impairment will have a clinical diagnosis of AD, ADRD, TBI, MCI, or other form of cognitive impairment. We will also be collecting data from caregivers as appropriate for sub-studies.

The second study cohort will feature individuals with a clinical diagnosis of DS; and/or their respective caregivers; who are using the MapHabit software in scope of MapHabit sub-studies. Subjects must be at least 5 years of age on the day of consent. No further demographic information regarding sex, gender, race, location, income, or living situation is defined at this time. This study will enroll individuals with a clinical diagnosis DS (n ~ 250) with the supervision of their parents/legal guardians.

## Inclusion Criteria

- **For Adult population with AD, ADRD, TBI, MCI, or other form of cognitive impairment:** Female or male subjects must be over the age of 18. A legally authorized representative (LAR) must sign on behalf of the subject if the subject does not have the capacity to consent
- **For Down Syndrome Population**: Females and males between 5-17 years of age. Parents or Legal guardians must sign for subjects under the age of 18.
- A clinical diagnosis of Alzheimer’s Disease (AD), Alzheimer’s Disease Related Dementia (ADRD), Mild Cognitive Impairment (MCI), Traumatic Brain Injury (TBI), Down Syndrome (DS) other forms of cognitive impairment, OR a caregiver of an individual with a diagnosis listed above
- Candidate participants with clinical diagnosis of AD/ADRD, MCI, TBI, or other form of cognitive impairment are screened for capacity to provide Informed Consent (see Section 10)
- Informed Consent obtained from candidate participant or legally authorized representative (LAR), parent or legal guardian as appropriate

## Exclusion Criteria

- Candidate participants with capacity and unwilling to provide Informed Consent for this study, or candidate participants without capacity and unable to provide Informed Consent through surrogacy, are excluded from enrollment

## Withdrawal Criteria

- Withdrawal of Informed Consent
- For caregivers of individuals with AD/ADRD, MCI, TBI, or other form of cognitive impairment: dissolution of a suitable patient-caregiver relationship

## Screen Failures

Subjects who are screened that do not meet inclusion criteria will not be enrolled in the trial.

# Study Methods

## Participants Joining the Umbrella Study from Sub-Study

All participants in any MapHabit, Inc. sub-study engaging with MapHabit software will be offered enrollment in this umbrella study at the time of enrollment to the respective sub-study. User data from participants not enrolled in this study will be handled according to protocols set forth by the MapHabit, Inc. sub-study(ies) the subject may be enrolled in. User data from subjects not enrolled in this umbrella study or subsequent sub-study(ies) will not be collected.

## Pre-recruitment

No pre-recruitment activities.

## Enrollment: Day 0

After creating the account, the subjects proceed to enrollment process, which consists of 2 steps:

1. **Inclusion/Exclusion Criteria Assessment and sub-study relevant pre-assessment questionnaires**
   1. Performed by trained and qualified study personnel
2. **Signing of Electronic Informed Consent Form (eICF) and HIPAA Waiver**

Please refer to the RESEARCH CONSENT FORM

## Data Collection Period

Participants will not take part in any assessment during the data collection period of the umbrella study. There is no predefined minimum data collection period. User interaction data will be passively collected during the time the participant engages with the software. For MapHabit sub-studies amongst the AD/RD population, participants and their caregivers will be expected to use the MapHabit System daily for 6 months. For MapHabit sub-studies amongst the DS population, children will need to use the MapHabit system daily for 3-6 months under the supervision of a parent or legal guardian. The maximum duration for passive user interaction data collection is two years. Parent(s) or legal guardians will be administered a periodic assessment on a monthly basis throughout the study in addition to the baseline assessments and the exit interviews for specific sub-studies that will occur at the end of the study period.

## Follow Up Assessments

No follow up assessments are included in this umbrella protocol. Participants of this study may undergo follow up assessments or questionnaires in accordance to subsequent MapHabit, Inc. sub-study(ies) the participant may be enrolled in. Samples of all assessments are included within this document. Baseline, Periodic, and Post Follow-up assessments for a sub-study amongst individuals with AD/RD may include:

- Quality of Life in Alzheimer’s Disease
- Zarit Burden Interview
- Pittsburgh Sleep Quality Index (PSQI)
- The Lawton Instrumental Activities of Daily Living Scale
- Neuropsychiatric Inventory (NPI) Questionnaire
- The Montreal Cognitive Assessment (MOCA)
- The Medication Management Instrument for Deficiencies in the Elderly (MedMaIDE)
- Geriatric Depression Scale
- Pain Assessment in Advanced Dementia Scale (PAINAD)
- Caregiver Self-Assessment Questionnaire
- MapHabit, Inc. Final Exit Interview Questionnaire
- Brief Interview for Mental Status – (BIMS) [Sample not included]

Baseline, Periodic and Post Follow-up assessments for a sub-study amongst individuals with DS may include:

- MapHabit, Inc Final Exit Interview Questionnaire
- LuMind Down Syndrome Caregiver Assessment
- Vineland-3
- Neuropsychiatric Inventory (NPI) Questionnaire
- Adaptive Behavior System Third Edition (ABAS-3) [Sample not included]
- Child Behavior Checklist (CBCL) [Sample not included]
- Behavior Problem Inventory (BPI-01)

# Statistical Considerations

No inferential statistical evaluation is foreseen in the scope of this study. Data will be listed and summarized by means of descriptive statistics in tabular and graphical formats.

## Sample Size Determination

Approximately 250 individuals with cognitive impairment and 250 caregivers of individuals with AD/RD will be enrolled in this umbrella study. Approximately 250 children with DS under the supervision of parents or legal guardians will be enrolled in this umbrella study. No formal sample size calculations were performed, as this is non-interventional, observational research. The number of participants was chosen based on feasibility and is considered sufficient to meet the study objectives and for potential sub-studies.

# Subject Accountability

Subjects who meet eligibility criteria and agree to participate in this study will be given an Informed Consent form, in writing or electronically, approved by an Institutional Review Board (IRB).

All subjects who meet eligibility criteria and endorse the Informed Consent form are considered enrolled in this study. Subjects enrolled in this study are followed in accordance to this umbrella protocol.

## Withdrawal

If a participant is withdrawn from this study, the reason(s) for withdrawal will be reported to the study data collection system. Data collected up to the point of withdrawal will be used for analysis and retained per protocol. No further user interaction data will be collected from the participant following their withdrawal.

# Safety

## Risk/Benefit Assessment

### Known Potential Risks

There are no known potential risks and/or discomforts associated with the passive collection of user interaction measures through software. For minors involved in the DS population, parental supervision and assent is required.

### Known Potential Benefits

Potential benefits are currently unknown. Data contributed as part of this study may benefit further development of the MapHabit software and research in cognitive impairment and may lead to development of diagnostic or therapeutic interventions in the future.

### Assessment of Potential Risks and Benefits

Since the study does not include an intervention, this protocol does not include any safety endpoints. When desired, study participants may revoke their consent.

## Unanticipated Problems

### Definition of Unanticipated Problems (UP)

The Office for Human Research Protections (OHRP) considers unanticipated problems involving risks to participants or others to include, in general, any incident, experience, or outcome that meets all of the following criteria:

- Unexpected in terms of nature, severity, or frequency given (a) the research procedures that are described in the protocol-related documents, such as the Institutional Review Board (IRB)-approved research protocol and Informed Consent document; and (b) the characteristics of the participant population being studied;
- Related or possibly related to participation in the research (“possibly related” means there is a reasonable possibility that the incident, experience, or outcome may have been caused by the procedures involved in the research); and
- Suggests that the research places participants or others at a greater risk of harm (including physical, psychological, economic, or social harm) than was previously known or recognized.

This definition could include an unanticipated adverse device effect, any serious adverse effects on health or safety or any life-threatening problem or death caused by, or associated with, a device, if that effect, problem, or death was not previously identified in nature, severity, or degree of incidence in the investigational plan or application (including a supplementary plan or application), or any other unanticipated serious problem associated with a device that relates to the rights, safety, or welfare of subjects (21 CFR 812.3(s)).

### Unanticipated Problem Reporting

The principal investigator (PI) will report unanticipated problems (UPs) to the selected commercial Institutional Review Board (IRB) and to the sponsor. The UP report will include the following information:

- Report date, IRB Study number, Study Title, Study Staff Contact Information, Date UP occurred, and date PI was notified about the UP.
- Description of the Unanticipated Problem which occurred during the conduct of the research.
- Provide an explanation for why this Unanticipated Problem occurred.
- Characterize the impact of the Unanticipated Problem on the study.
- Describe the steps which have been taken to resolve the reported occurrence.
- Describe the plan implemented to avoid or prevent future occurrences.
- Inform other study participants as necessary.
- Name all other entities to which this UP has been reported.
- Determine if the UP will require modification of the currently approved study and/or consent form.

Technical issues occurring during the application use can be reported via email support@maphabit.com.

### Serious Adverse Event (SAE) Reporting

A Serious Adverse Event (SAE) is an untoward occurrence in human research that results in death, a life-threatening experience, inpatient hospitalization, prolongation of hospitalization, persistent or significant disability or incapacity, congenital anomaly, or birth defect, or that requires medical, surgical, behavioral, social, or other intervention to prevent such an outcome (21 CFR 312.32(a)).

Adverse events and deaths occurring in the course of an approved study that are serious, unanticipated and related or probably related to the research, by the judgment of the investigator, will be reported to the IRB.

If the event satisfies ALL three of these criteria the event must be reported to the IRB within 5 business days of learning of the event. The study sponsor will also be notified within the same time-frame.

### Adverse Device Event Reporting

An investigator shall submit to the sponsor and to the reviewing Institutional Review Board (IRB) a report of any unanticipated adverse device effect occurring during an investigation as soon as possible, but in no event later than 10 working days after the investigator first learns of the effect (21 CFR 812.150(a)(1)), A sponsor who conducts an evaluation of an unanticipated adverse device effect under 812.46(b) shall report the results of such evaluation to the Food and Drug Administration (FDA) and to all reviewing IRB's and participating investigators within 10 working days after the sponsor first receives notice of the effect. Thereafter the sponsor shall submit such additional reports concerning the effect as FDA requests (21 CFR 812.150(b)(1)).

## Death Reporting

Death is reported as is stated in section 9.2.3 Serious Adverse Event (SAE) Reporting.

A death that meets all three criteria for an SAE, as above, shall be reported by Study Staff to the IRB within 5 business days of learning of the death. A detailed narrative or death letter may be requested by the sponsor including date of death, place, circumstances, cause of death, and whether the death was witnessed or not.

# Informed Consent

Subject participation in this clinical study is voluntary. Informed Consent is required from each subject. The Investigator is responsible for ensuring that Informed Consent is obtained prior to the use of any investigational devices, study-required procedures and/or testing, or data collection. Informed Consent in this study will be obtained in writing during a face-to-face encounter with the study investigator or clinical coordinator, or electronically through a website or within the MapHabit software. For participants under the age of 18, a parent of legal guardian must sign on behalf of the participant. An assent form for DS participants will be provided to inform and teach about the significance of the sub-study participants that are minors.

The obtaining and documentation of Informed Consent must be in accordance with the principles of the Declaration of Helsinki, ISO 14155, any applicable national regulations, and local IRB. The ICF must be accepted by the sponsor and approved by the IRB.

The process of obtaining Informed Consent shall at a minimum include the following steps, as well as any other steps required by applicable laws, rules, regulations and guidelines:

- include a description of all aspects of the clinical study that are relevant to the subject’s decision to participate throughout the clinical study,
- avoid any coercion of or undue influence of subjects to participate,
- not waive or appear to waive subject’s legal rights,
- use native language that is non-technical and understandable to the subject or his/her legal representative,
- provide ample time for the subject to consider participation and ask questions if necessary, ensure important new information is provided to new and existing subjects throughout the clinical study.

Consent forms describing in detail the study intervention, study procedures, and risks are given to the participant and written documentation of Informed Consent is required prior to starting intervention/administering study intervention.

## Assessment of Capacity for Informed Consent

All candidate participants with a clinical diagnosis of AD/ADRD, MCI, TBI, or other form of cognitive impairment are screened using the University of California San Diego Brief Assessment of Capacity to Consent (UBACC) [5]. The UBACC has a 10-item scale, including questions that focus on understanding and appreciation of the disclosed research information. In cases where screening with the UBACC suggests that a candidate participant may lack adequate capacity to consent, Informed Consent may be gathered from a legally authorized representative. All research staff administering the UBACC will undergo training to gain familiarity with the UBACC scoring system prior to administering the screening test.

## Process of Obtaining Informed Consent Through Surrogacy

Informed Consent may be gathered from a legally authorized representative (LAR), parent, or legal guardian in cases where screening suggests that a candidate participant may lack adequate capacity to consent or is under the age of 18. In such cases, the consent interview with the LAR may be conducted in-person or over the phone. In cases where the consent interview is conducted by phone, a copy of the RESEARCH CONSENT FORM is first sent to the LAR, parent, or legal guardian by email or other means for review, prior to the consent interview. Informed Consent is considered obtained after the consent interview has occurred, and a signed and dated copy of the RESEARCH CONSENT FORM is returned from the LAR, parent, or legal guardian to the study coordinator.

# Protocol Deviations

A protocol deviation is any noncompliance with the umbrella trial protocol. It is the responsibility of the investigator to use continuous vigilance to identify deviations and report them within 3 working days of identification of the protocol deviation, or within 3 working days of the scheduled protocol-required activity. All deviations must be addressed in study source documents. Protocol deviations must be sent to the reviewing Institutional Review Board (IRB) per their policies. The study investigator is responsible for knowing and adhering to the reviewing IRB requirements. Protocol deviations must also be reported in the study data collection system.

# Study Discontinuation and Closure

This study may be temporarily suspended or prematurely terminated if there is sufficient reasonable cause. Written notification, documenting the reason for study suspension or termination, will be provided by suspending or terminating party to study participants, investigator, sponsor and regulatory authorities. If the study is prematurely terminated or suspended, the Principal Investigator (PI) will promptly inform study participants, the Institutional Review Board (IRB), and sponsor and will provide the reason(s) for the termination or suspension. Study participants will be contacted via phone or email, and be informed of changes to study schedule.

Circumstances that may warrant termination or suspension include, but are not limited to:

- Determination of unexpected, significant, or unacceptable risk to participants
- Demonstration of efficacy that would warrant stopping
- Insufficient compliance to protocol requirements
- Data that are not sufficiently complete and/or evaluable
- Determination that the primary endpoint has been met
- Determination of futility

Study may resume once concerns about safety, protocol compliance, and data quality are addressed, and satisfy the sponsor, IRB and/or Food and Drug Administration (FDA).

# Data Management

## Data analysis plan

Data will be listed and summarized by means of descriptive statistics in tabular and graphical formats.

## Quality control

All research personnel will complete CITI training and be knowledgeable about Good Clinical Practice (GCP), relevant regulations and policies. The Principal Investigator will ensure adherence to the study protocol, regulatory requirements, GCP standards at all times. Team meeting will be held on a regular basis to review adherence to study protocol, and to discuss any incidents of failures of protocol compliance, keeping adequate and accurate records, and reporting of adverse events. These outcomes of these meetings will be reported to the sponsor and to applicable regulatory bodies as requested.

## Data handling

The final storage space for the study data will be a cloud database and external file storage system.

The database contains a table with the participant codes ("Participant ID", or "userID") assigned to them in the application, and their respective:

- Screen views: for each screen, time during which the screen was displayed to the user
- Interactions with all in-app actionable elements (such as buttons) with the element identifier and the time of such interaction.
- Application logs, such as error reports, unexpected shutdowns, etc.

Participant ID (or UserID) will not incorporate personally identifiable information.

All electronic files will be password-protected and stored on a commercial secured and encrypted server and will only be accessible to authorized research personnel. All access will be audit logged. As data will exist in de-identified form, the electronic data files may be kept indefinitely, if not stated otherwise in a subsequent MapHabit sub-study. The Principal Investigator will be responsible should receipt and/or transmission of data be required. All data transported/shared will be de-identified.

## Data Collection

Data will be collected passively using the Google Firebase Analytics platform and will be securely transmitted from the application to the sponsor-maintained cloud database.

## Study Records Retention

All Protocol Files related to the review and oversight of human subjects’ research protocols submitted by the research team to the IRB shall have a cutoff at the end of the fiscal year after the research project has been completed or terminated. Records will be retained for 10 years after the cutoff.

Records include, but are not limited to, the application to the IRB; research protocol and amendments; case reports forms; Informed Consent template and HIPAA Authorization template; reports of adverse events, complaints, and deviations from IRB-approved protocol; data and safety monitoring reports; research findings to date; and all relevant documents and related correspondences between the IRB and the investigators in the review of an associated protocol.

No records will be destroyed without the written consent of the sponsor, if applicable. It is the responsibility of the Study Staff to inform the sponsor when these documents no longer need to be retained.

# Abbreviations

| **Abbreviation** | **Description** |
| --- | --- |
| **AD** | Alzheimer’s Dementia |
| **ADL’s** | Activities of Daily Living |
| **ADRD** | Alzheimer’s Dementia Related Disease |
| **ADE** | Adverse Events |
| **CITI** | Collaborative Institutional Training Initiative |
| **eICF** | Electronic Informed Consent Form |
| **FDA** | Food and Drug Administration |
| **GCP** | Good Clinical Practice |
| **HIPAA** | Health Insurance Portability and Accountability Act of 1996 |
| **ICF** | Informed Consent Form |
| **IRB** | Institutional Review Board |
| **MCI** | Mild Cognitive Impairment |
| **PI** | Principal Investigator |
| **QoL** | Quality of Life |
| **SAE** | Serious Adverse Event |
| **TBI** | Traumatic Brain Injury |
| **UP** | Unanticipated Problem |

# Statement of Compliance

The trial will be conducted in accordance with International Conference on Harmonization Good Clinical Practice (ICH GCP) and applicable United States (US) Code of Federal Regulations (CFR). The Principal Investigator will assure that no deviation from, or changes to the protocol will take place without prior agreement from the sponsor and documented approval from the Institutional Review Board (IRB), except where necessary to eliminate an immediate hazard(s) to the trial participants.

The protocol, Informed Consent form(s), recruitment materials, and all participant materials will be submitted to the IRB for review and approval. Approval of both the protocol and the consent form must be obtained before any participant is enrolled. Any amendment to the protocol will require review and approval by the IRB before the changes are implemented to the study. All changes to the consent form will be IRB approved; a determination will be made regarding whether a new consent needs to be obtained from participants who provided consent, using a previously approved consent form.

16. Potential Sub-Study Surveys & Questionnaires

- 1. Quality of Life in Alzheimer’s Disease
  2. The Zarit Burden Interview
  3. The Pittsburgh Sleep Quality Index (PSQI)
  4. The Lawton Instrumental Activities of Daily Living Scale
  5. The Neuropsychiatric Inventory Questionnaire
  6. The Montreal Cognitive Assessment (MOCA)
  7. Live Alone Assessment Adapted from the University of Iowa
  8. Medication Management Instrument for Deficiencies in the Elderly (MedMaIDE^TM^)
  9. Geriatric Depression Scale
  10. Pain Assessment in Advanced Dementia Scale (PAINAD)
  11. Caregiver Self-Assessment Questionnaire
  12. LuMind Down Syndrome Caregiver Assessment

**Caregiver Survey on Independence in Down Syndrome**

1. Contact Information
   1. Name: ____________
   2. Company: ____________
   3. Address: ____________
   4. Address 2: ____________
   5. City/Town: ____________
   6. State Province (If in the United States): ____________
   7. ZIP/Postal Code: ____________
   8. Country: ____________
   9. Email Address: ____________
   10. Phone Number: ____________
2. Who is completing this survey? Please select all options below that best describes your relationship to the loved on with Down Syndrome
   1. Parent
   2. Sibling
   3. Other Caregiver
   4. Other (Please specify)
3. Your age?
   1. Under 18
   2. 18-24
   3. 25-34
   4. 35-44
   5. 45-54
   6. 55-64
   7. 65+
4. Highest Education Level
   1. Elementary School
   2. Middle School
   3. High School Graduate
   4. College Graduate
   5. Post-Graduate Degree
5. What country do you live in?
   1. ____________
6. In which town or city do you live in?
   1. ____________
7. If in the U.S., which U.S. state do you live in?
   1. ____________
8. What is your zip code (if you live in the U.S.) or postal code (if you live outside the U.S.)?
   1. ____________
9. What is your approximate average household income?
   1. Under $49,999 (Under R44,730)
   2. Between $50,000 and $99,999 (Between €44,731 - 89,463)
   3. Between $100,000 and $149,999 (Between €89,464 - 134,195)
   4. Between $150,000 and $199,999 (Between €134,196 - 178,927)
   5. Over $200,000 (Over 178,928)
   6. Prefer not to answer.
10. What was the date of birth of the loved one with Down Syndrome?
    1. ____________
11. Does the loved one with Down syndrome live in the location listed previously?
    1. Yes
    2. No
12. What country does the loved one with Down syndrome live in?
    1. ____________
13. In which town or city does the loved one with Down syndrome live in?
    1. ____________
14. If in the US, which state does the person with Down syndrome live in?
    1. ____________
15. What is the loved one with Down syndrome zip code (if they live in the United States) or postal code (if they live outside the US?)
    1. ____________
16. Does the loved one with Down syndrome live:
    1. Independently
    2. In a group home
    3. With family
    4. Other (please specify) ____________
17. Which race/ethnicity best describes the loved one with Down Syndrome? (Please choose)
    1. White or Caucasian
    2. Black or African American
    3. Hispanic or Latinx
    4. Asian or Asian American
    5. Native American or Alaska Native
    6. Native Hawaiian or other Pacific Islander
    7. Another race
18. What is the loved one with Down syndrome's gender?
    1. Female
    2. Male
    3. Non-Binary
    4. Other
19. My loved one with Down syndrome has an additional diagnosis, which has been confirmed by a medical professional, (choose all that apply) of:
    1. Attention Deficit Hyperactivity Disorder (ADHD)
    2. Autism or Autism Spectrum Disorder (ASD)
    3. Regression
    4. Sleep Apnea
    5. Celiac Disease
    6. Obsessive Compulsive Disorder (OCD)
    7. Mental Health diagnosis
    8. Dementia or Alzheimer's Disease
    9. Crohn's Disease
    10. Moya Moya
    11. Obesity
    12. Medically Complex
    13. Mobility (Please describe below)
        1. ____________
    14. None of the above
    15. Other diagnosis that you think affects the loved one with Down syndrome's (Please specify)
        1. ___________
20. I feel the following **SAFETY** topics are important for my loved one with Down syndrome (Check all that apply):
    1. Social media safety
       1. Achieved already
       2. Not achieved but not important
       3. No achieved but important to me now
       4. Not achieved but may be important to me in the future
       5. Don’t know
    2. Internet safety
       1. Achieved already
       2. Not achieved but not important
       3. No achieved but important to me now
       4. Not achieved but may be important to me in the future
       5. Don’t know
    3. Bullying
       1. Achieved already
       2. Not achieved but not important
       3. No achieved but important to me now
       4. Not achieved but may be important to me in the future
       5. Don’t know
    4. Sexual Abuse
       1. Achieved already
       2. Not achieved but not important
       3. No achieved but important to me now
       4. Not achieved but may be important to me in the future
       5. Don’t know
    5. Verbal Abuse
       1. Achieved already
       2. Not achieved but not important
       3. No achieved but important to me now
       4. Not achieved but may be important to me in the future
       5. Don’t know
    6. Stranger Danger
       1. Achieved already
       2. Not achieved but not important
       3. No achieved but important to me now
       4. Not achieved but may be important to me in the future
       5. Don’t know
    7. Safely crossing a street/parking lot
       1. Achieved already
       2. Not achieved but not important
       3. No achieved but important to me now
       4. Not achieved but may be important to me in the future
       5. Don’t know
    8. Bolting/Elopement (running away from caregiver)
       1. Achieved already
       2. Not achieved but not important
       3. No achieved but important to me now
       4. Not achieved but may be important to me in the future
       5. Don’t know
    9. Kitchen/cooking safety
       1. Achieved already
       2. Not achieved but not important
       3. No achieved but important to me now
       4. Not achieved but may be important to me in the future
       5. Don’t know
    10. Water safety
        1. Achieved already
        2. Not achieved but not important
        3. No achieved but important to me now
        4. Not achieved but may be important to me in the future
        5. Don’t know
    11. Staying with a babysitter or other caregiver
        1. Achieved already
        2. Not achieved but not important
        3. No achieved but important to me now
        4. Not achieved but may be important to me in the future
        5. Don’t know
    12. Left home alone for greater than 2 hours
        1. Achieved already
        2. Not achieved but not important
        3. No achieved but important to me now
        4. Not achieved but may be important to me in the future
        5. Don’t know
    13. Go out alone
        1. Achieved already
        2. Not achieved but not important
        3. No achieved but important to me now
        4. Not achieved but may be important to me in the future
        5. Don’t know
    14. Other (please explain): _____________
        1. Achieved already
        2. Not achieved but not important
        3. No achieved but important to me now
        4. Not achieved but may be important to me in the future
        5. Don’t know
21. Top 3 choices for **SAFETY** from question 20
    1. 1^st^ Choice: ________
    2. 2^nd^ Choice:________
    3. 3^rd^ Choice:________
22. I feel the following SELF-CARE topics are important for my loved one with Down syndrome:
    1. Dress and undress self
       1. Achieved already
       2. Not achieved but not important
       3. No achieved but important to me now
       4. Not achieved but may be important to me in the future
       5. Don’t know
    2. Putting on/tying shoes
       1. Achieved already
       2. Not achieved but not important
       3. No achieved but important to me now
       4. Not achieved but may be important to me in the future
       5. Don’t know
    3. Use toilet independently
       1. Achieved already
       2. Not achieved but not important
       3. No achieved but important to me now
       4. Not achieved but may be important to me in the future
       5. Don’t know
    4. Grooming, brushing teeth, combing and/or brushing hair
       1. Achieved already
       2. Not achieved but not important
       3. No achieved but important to me now
       4. Not achieved but may be important to me in the future
       5. Don’t know
    5. Wash himself/herself (shower or bath)
       1. Achieved already
       2. Not achieved but not important
       3. No achieved but important to me now
       4. Not achieved but may be important to me in the future
       5. Don’t know
    6. Shaving
       1. Achieved already
       2. Not achieved but not important
       3. No achieved but important to me now
       4. Not achieved but may be important to me in the future
       5. Don’t know
    7. Cutting fingernails and toenails
       1. Achieved already
       2. Not achieved but not important
       3. No achieved but important to me now
       4. Not achieved but may be important to me in the future
       5. Don’t know
    8. Female hygiene needs
       1. Achieved already
       2. Not achieved but not important
       3. No achieved but important to me now
       4. Not achieved but may be important to me in the future
       5. Don’t know
    9. Healthy eating/portion control
       1. Achieved already
       2. Not achieved but not important
       3. No achieved but important to me now
       4. Not achieved but may be important to me in the future
       5. Don’t know
    10. Take own medications
        1. Achieved already
        2. Not achieved but not important
        3. No achieved but important to me now
        4. Not achieved but may be important to me in the future
        5. Don’t know
    11. Self-reporting feelings and health
        1. Achieved already
        2. Not achieved but not important
        3. No achieved but important to me now
        4. Not achieved but may be important to me in the future
        5. Don’t know
    12. Understanding of puberty
        1. Achieved already
        2. Not achieved but not important
        3. No achieved but important to me now
        4. Not achieved but may be important to me in the future
        5. Don’t know
    13. Healthy understanding of sexuality
        1. Achieved already
        2. Not achieved but not important
        3. No achieved but important to me now
        4. Not achieved but may be important to me in the future
        5. Don’t know
    14. Someone "safe" to talk to about thoughts and feelings
        1. Achieved already
        2. Not achieved but not important
        3. No achieved but important to me now
        4. Not achieved but may be important to me in the future
        5. Don’t know
    15. Other (please explain)
        1. Achieved already
        2. Not achieved but not important
        3. No achieved but important to me now
        4. Not achieved but may be important to me in the future
        5. Don’t know
23. Top 3 choices for **SELF-CARE** from question 22:
    1. 1^st^ Choice: ________
    2. 2^nd^ Choice: ________
    3. 3^rd^ Choice: ________
24. I feel the following **DAILY LIVING** topics are important for my loved one with Down syndrome:
    1. Drink independently from a cup
       1. Achieved already
       2. Not achieved but not important
       3. No achieved but important to me now
       4. Not achieved but may be important to me in the future
       5. Don’t know
    2. Eat meals independently
       1. Achieved already
       2. Not achieved but not important
       3. No achieved but important to me now
       4. Not achieved but may be important to me in the future
       5. Don’t know
    3. Move around in or out of the house
       1. Achieved already
       2. Not achieved but not important
       3. No achieved but important to me now
       4. Not achieved but may be important to me in the future
       5. Don’t know
    4. Navigating public transportation alone
       1. Achieved already
       2. Not achieved but not important
       3. No achieved but important to me now
       4. Not achieved but may be important to me in the future
       5. Don’t know
    5. Traveling alone (on planes, trains, etc.)
       1. Achieved already
       2. Not achieved but not important
       3. No achieved but important to me now
       4. Not achieved but may be important to me in the future
       5. Don’t know
    6. Living independently/semi-independently
       1. Achieved already
       2. Not achieved but not important
       3. No achieved but important to me now
       4. Not achieved but may be important to me in the future
       5. Don’t know
    7. Driving
       1. Achieved already
       2. Not achieved but not important
       3. No achieved but important to me now
       4. Not achieved but may be important to me in the future
       5. Don’t know
    8. Doing errands, including shopping in stores
       1. Achieved already
       2. Not achieved but not important
       3. No achieved but important to me now
       4. Not achieved but may be important to me in the future
       5. Don’t know
    9. Use money
       1. Achieved already
       2. Not achieved but not important
       3. No achieved but important to me now
       4. Not achieved but may be important to me in the future
       5. Don’t know
    10. Manage daily finances (keep track of cash, checking account, pay bills, etc.)
        1. Achieved already
        2. Not achieved but not important
        3. No achieved but important to me now
        4. Not achieved but may be important to me in the future
        5. Don’t know
    11. Carry out domestic activities (make bed, pick up around the house, light housecleaning, etc.)
        1. Achieved already
        2. Not achieved but not important
        3. No achieved but important to me now
        4. Not achieved but may be important to me in the future
        5. Don’t know
    12. Doing laundry, washing and drying
        1. Achieved already
        2. Not achieved but not important
        3. No achieved but important to me now
        4. Not achieved but may be important to me in the future
        5. Don’t know
    13. Use a watch
        1. Achieved already
        2. Not achieved but not important
        3. No achieved but important to me now
        4. Not achieved but may be important to me in the future
        5. Don’t know
    14. Follow a schedule
        1. Achieved already
        2. Not achieved but not important
        3. No achieved but important to me now
        4. Not achieved but may be important to me in the future
        5. Don’t know
    15. Time Management
        1. Achieved already
        2. Not achieved but not important
        3. No achieved but important to me now
        4. Not achieved but may be important to me in the future
        5. Don’t know
    16. Prepare simple meals (requiring no mixing or cooking, including sandwiches, cold cereal, etc.)
        1. Achieved already
        2. Not achieved but not important
        3. No achieved but important to me now
        4. Not achieved but may be important to me in the future
        5. Don’t know
    17. Cook meals (fry eggs, make pancakes, etc.)
        1. Achieved already
        2. Not achieved but not important
        3. No achieved but important to me now
        4. Not achieved but may be important to me in the future
        5. Don’t know
    18. Weight Management
        1. Achieved already
        2. Not achieved but not important
        3. No achieved but important to me now
        4. Not achieved but may be important to me in the future
        5. Don’t know
    19. Other (please explain)
        1. Achieved already
        2. Not achieved but not important
        3. No achieved but important to me now
        4. Not achieved but may be important to me in the future
        5. Don’t know
25. Top 3 choices for **DAILY LIVING** from question 24:
    1. 1^st^ Choice: ________
    2. 2^nd^ Choice: ________
    3. 3^rd^ Choice: ________
26. I feel the following **SOCIAL/LEISURE TIME** topics are important for my loved one with Down syndrome:
    1. Engages in leisure time appropriately
       1. Achieved already
       2. Not achieved but not important
       3. No achieved but important to me now
       4. Not achieved but may be important to me in the future
       5. Don’t know
    2. Able to manage disruptions to routines or schedules
       1. Achieved already
       2. Not achieved but not important
       3. No achieved but important to me now
       4. Not achieved but may be important to me in the future
       5. Don’t know
    3. Watching TV
       1. Achieved already
       2. Not achieved but not important
       3. No achieved but important to me now
       4. Not achieved but may be important to me in the future
       5. Don’t know
    4. Listening to music
       1. Achieved already
       2. Not achieved but not important
       3. No achieved but important to me now
       4. Not achieved but may be important to me in the future
       5. Don’t know
    5. Volunteering
       1. Achieved already
       2. Not achieved but not important
       3. No achieved but important to me now
       4. Not achieved but may be important to me in the future
       5. Don’t know
    6. Playing games (card games)
       1. Achieved already
       2. Not achieved but not important
       3. No achieved but important to me now
       4. Not achieved but may be important to me in the future
       5. Don’t know
    7. Participate in hobbies (painting, gardening, writing, dancing, etc.)
       1. Achieved already
       2. Not achieved but not important
       3. No achieved but important to me now
       4. Not achieved but may be important to me in the future
       5. Don’t know
    8. Sports activities
       1. Achieved already
       2. Not achieved but not important
       3. No achieved but important to me now
       4. Not achieved but may be important to me in the future
       5. Don’t know
    9. Walking
       1. Achieved already
       2. Not achieved but not important
       3. No achieved but important to me now
       4. Not achieved but may be important to me in the future
       5. Don’t know
    10. Hanging out with friends
        1. Achieved already
        2. Not achieved but not important
        3. No achieved but important to me now
        4. Not achieved but may be important to me in the future
        5. Don’t know
    11. Going out to eat
        1. Achieved already
        2. Not achieved but not important
        3. No achieved but important to me now
        4. Not achieved but may be important to me in the future
        5. Don’t know
    12. Religious activities (church, etc.)
        1. Achieved already
        2. Not achieved but not important
        3. No achieved but important to me now
        4. Not achieved but may be important to me in the future
        5. Don’t know
    13. Going to the movies
        1. Achieved already
        2. Not achieved but not important
        3. No achieved but important to me now
        4. Not achieved but may be important to me in the future
        5. Don’t know
    14. Other (please explain)
        1. Achieved already
        2. Not achieved but not important
        3. No achieved but important to me now
        4. Not achieved but may be important to me in the future
        5. Don’t know
27. Top 3 choices for **SOCIAL/LEISURE TIME** from question 26:
    1. 1^st^ Choice: ________
    2. 2^nd^ Choice: ________
    3. 3^rd^ Choice: ________
28. I feel the following **VOCATIONAL/EMPLOYMENT** topics are important for my loved one with Down syndrome:
    1. Reading and writing
       1. Achieved already
       2. Not achieved but not important
       3. No achieved but important to me now
       4. Not achieved but may be important to me in the future
       5. Don’t know
    2. Driving
       1. Achieved already
       2. Not achieved but not important
       3. No achieved but important to me now
       4. Not achieved but may be important to me in the future
       5. Don’t know
    3. Navigating public transportation alone
       1. Achieved already
       2. Not achieved but not important
       3. No achieved but important to me now
       4. Not achieved but may be important to me in the future
       5. Don’t know
    4. Traveling alone (on planes, trains, etc.)
       1. Achieved already
       2. Not achieved but not important
       3. No achieved but important to me now
       4. Not achieved but may be important to me in the future
       5. Don’t know
    5. Time management
       1. Achieved already
       2. Not achieved but not important
       3. No achieved but important to me now
       4. Not achieved but may be important to me in the future
       5. Don’t know
    6. Interpersonal skills
       1. Achieved already
       2. Not achieved but not important
       3. No achieved but important to me now
       4. Not achieved but may be important to me in the future
       5. Don’t know
    7. Money management
       1. Achieved already
       2. Not achieved but not important
       3. No achieved but important to me now
       4. Not achieved but may be important to me in the future
       5. Don’t know
    8. Ability to ask for help when needed
       1. Achieved already
       2. Not achieved but not important
       3. No achieved but important to me now
       4. Not achieved but may be important to me in the future
       5. Don’t know
    9. Respecting personal boundaries
       1. Achieved already
       2. Not achieved but not important
       3. No achieved but important to me now
       4. Not achieved but may be important to me in the future
       5. Don’t know
    10. Appropriate behavior
        1. Achieved already
        2. Not achieved but not important
        3. No achieved but important to me now
        4. Not achieved but may be important to me in the future
        5. Don’t know
    11. Ability to focus on task at hand
        1. Achieved already
        2. Not achieved but not important
        3. No achieved but important to me now
        4. Not achieved but may be important to me in the future
        5. Don’t know
    12. Organization skills
        1. Achieved already
        2. Not achieved but not important
        3. No achieved but important to me now
        4. Not achieved but may be important to me in the future
        5. Don’t know
    13. Understand what he/she reads
        1. Achieved already
        2. Not achieved but not important
        3. No achieved but important to me now
        4. Not achieved but may be important to me in the future
        5. Don’t know
    14. Use verbal communication
        1. Achieved already
        2. Not achieved but not important
        3. No achieved but important to me now
        4. Not achieved but may be important to me in the future
        5. Don’t know
    15. Be understood by others, even with limited language
        1. Achieved already
        2. Not achieved but not important
        3. No achieved but important to me now
        4. Not achieved but may be important to me in the future
        5. Don’t know
    16. Use telephone
        1. Achieved already
        2. Not achieved but not important
        3. No achieved but important to me now
        4. Not achieved but may be important to me in the future
        5. Don’t know
    17. Use a computer for internet and e-mail
        1. Achieved already
        2. Not achieved but not important
        3. No achieved but important to me now
        4. Not achieved but may be important to me in the future
        5. Don’t know
    18. Other (please explain)
        1. Achieved already
        2. Not achieved but not important
        3. No achieved but important to me now
        4. Not achieved but may be important to me in the future
        5. Don’t know
29. Top 3 choices for **VOCATIONAL/EMPLOYMENT** from question 28:
    1. 1^st^ Choice: ________
    2. 2^nd^ Choice: ________
    3. 3^rd^ Choice: ________
30. I feel the following **COMMUNICATION TOPICS** are important for my loved one with Down syndrome:
    1. Communicating wants and needs
       1. Achieved already
       2. Not achieved but not important
       3. No achieved but important to me now
       4. Not achieved but may be important to me in the future
       5. Don’t know
    2. Browse picture books without reading
       1. Achieved already
       2. Not achieved but not important
       3. No achieved but important to me now
       4. Not achieved but may be important to me in the future
       5. Don’t know
    3. Write his/her own name
       1. Achieved already
       2. Not achieved but not important
       3. No achieved but important to me now
       4. Not achieved but may be important to me in the future
       5. Don’t know
    4. Understand what he/she writes
       1. Achieved already
       2. Not achieved but not important
       3. No achieved but important to me now
       4. Not achieved but may be important to me in the future
       5. Don’t know
    5. Comprehend reading aloud
       1. Achieved already
       2. Not achieved but not important
       3. No achieved but important to me now
       4. Not achieved but may be important to me in the future
       5. Don’t know
    6. Write to communicate
       1. Achieved already
       2. Not achieved but not important
       3. No achieved but important to me now
       4. Not achieved but may be important to me in the future
       5. Don’t know
    7. Read for pleasure
       1. Achieved already
       2. Not achieved but not important
       3. No achieved but important to me now
       4. Not achieved but may be important to me in the future
       5. Don’t know
    8. Understand what he/she reads
       1. Achieved already
       2. Not achieved but not important
       3. No achieved but important to me now
       4. Not achieved but may be important to me in the future
       5. Don’t know
    9. Use verbal communication
       1. Achieved already
       2. Not achieved but not important
       3. No achieved but important to me now
       4. Not achieved but may be important to me in the future
       5. Don’t know
    10. Be understood by others, even with limited language
        1. Achieved already
        2. Not achieved but not important
        3. No achieved but important to me now
        4. Not achieved but may be important to me in the future
        5. Don’t know
    11. Use telephone
        1. Achieved already
        2. Not achieved but not important
        3. No achieved but important to me now
        4. Not achieved but may be important to me in the future
        5. Don’t know
    12. Use computer to play
        1. Achieved already
        2. Not achieved but not important
        3. No achieved but important to me now
        4. Not achieved but may be important to me in the future
        5. Don’t know
    13. Use a computer for internet and e-mail
        1. Achieved already
        2. Not achieved but not important
        3. No achieved but important to me now
        4. Not achieved but may be important to me in the future
        5. Don’t know
    14. Sharing personal information appropriately
        1. Achieved already
        2. Not achieved but not important
        3. No achieved but important to me now
        4. Not achieved but may be important to me in the future
        5. Don’t know
    15. Getting to/Making/Asking questions at doctor appointments
        1. Achieved already
        2. Not achieved but not important
        3. No achieved but important to me now
        4. Not achieved but may be important to me in the future
        5. Don’t know
    16. Ability to consent to medical treatment
        1. Achieved already
        2. Not achieved but not important
        3. No achieved but important to me now
        4. Not achieved but may be important to me in the future
        5. Don’t know
    17. Able to verbalize, sign or use assistive communication device to express wants and needs
        1. Achieved already
        2. Not achieved but not important
        3. No achieved but important to me now
        4. Not achieved but may be important to me in the future
        5. Don’t know
    18. Expresses emotions appropriately
        1. Achieved already
        2. Not achieved but not important
        3. No achieved but important to me now
        4. Not achieved but may be important to me in the future
        5. Don’t know
    19. Ability to participate in a conversation (verbal, sign language or via a communication devise)
        1. Achieved already
        2. Not achieved but not important
        3. No achieved but important to me now
        4. Not achieved but may be important to me in the future
        5. Don’t know
    20. Other (please explain): _______________
        1. Achieved already
        2. Not achieved but not important
        3. No achieved but important to me now
        4. Not achieved but may be important to me in the future
        5. Don’t know
31. Top 3 choices for **VOCATIONAL/EMPLOYMENT** from question 30:
    1. 1^st^ Choice: ________
    2. 2^nd^ Choice: ________
    3. 3^rd^ Choice: ________
32. Which statement below best describes your wishes as a **CAREGIVER** for your loved one with Down syndrome?
    1. Be as independent as possible
    2. Achieve some independence
    3. Independence is not a priority
33. The following **CAREGIVER** self-care topics are important to me as the caregiver of a loved one with Down syndrome in terms of MY independence. This question is about YOUR ability as a caregiver to be independent of your loved one with Down syndrome.
    1. Run errands with loved one safely at home
       1. Achieved already
       2. Not achieved but not important
       3. No achieved but important to me now
       4. Not achieved but may be important to me in the future
       5. Don’t know
    2. Run errands with loved one safely at my side
       1. Achieved already
       2. Not achieved but not important
       3. No achieved but important to me now
       4. Not achieved but may be important to me in the future
       5. Don’t know
    3. Vacations
       1. Achieved already
       2. Not achieved but not important
       3. No achieved but important to me now
       4. Not achieved but may be important to me in the future
       5. Don’t know
    4. Respite care
       1. Achieved already
       2. Not achieved but not important
       3. No achieved but important to me now
       4. Not achieved but may be important to me in the future
       5. Don’t know
    5. Financial planning
       1. Achieved already
       2. Not achieved but not important
       3. No achieved but important to me now
       4. Not achieved but may be important to me in the future
       5. Don’t know
    6. Ability to care for self
       1. Achieved already
       2. Not achieved but not important
       3. No achieved but important to me now
       4. Not achieved but may be important to me in the future
       5. Don’t know
    7. Ability to call for emergency at appropriate times
       1. Achieved already
       2. Not achieved but not important
       3. No achieved but important to me now
       4. Not achieved but may be important to me in the future
       5. Don’t know
    8. Ability to know how to get help
       1. Achieved already
       2. Not achieved but not important
       3. No achieved but important to me now
       4. Not achieved but may be important to me in the future
       5. Don’t know
    9. Understanding entitlement programs such as Medicaid and Social Security
       1. Achieved already
       2. Not achieved but not important
       3. No achieved but important to me now
       4. Not achieved but may be important to me in the future
       5. Don’t know
    10. What will happen after I am gone
        1. Achieved already
        2. Not achieved but not important
        3. No achieved but important to me now
        4. Not achieved but may be important to me in the future
        5. Don’t know
    11. Burden of multiple medical appointments
        1. Achieved already
        2. Not achieved but not important
        3. No achieved but important to me now
        4. Not achieved but may be important to me in the future
        5. Don’t know
    12. Reduce stress of the planning for my loved one with DS’s options after age 22
        1. Achieved already
        2. Not achieved but not important
        3. No achieved but important to me now
        4. Not achieved but may be important to me in the future
        5. Don’t know
    13. Other (please explain)
        1. Achieved already
        2. Not achieved but not important
        3. No achieved but important to me now
        4. Not achieved but may be important to me in the future
        5. Don’t know
34. Top 3 choices for **CAREGIVER** self-care from question 33:
    1. 1^st^ Choice: ________
    2. 2^nd^ Choice: ________
    3. 3^rd^ Choice: ________
35. Do you have ideas on further research into practical solutions for supporting greater independence?
    1. No
    2. Yes (please explain) ___________________
    3. Vineland-3 Adaptive Behavior Scales
    4. Behavior Problems Inventory
    5. MapHabit, Inc. Final Exit Interview Questionnaire

Date: ___________________ Name or ID: __________________________

Compared to three months ago, before you and ___(name)___ started using the MapHabit System:

- Are you now experiencing:
- Is ___(name)___ now experiencing:

Please use the scale indicated

1 = Much negative change 2 = Some negative change 3 = No change 4 = Some positive change 5 = Much positive change

1. Improved mood
2. Improved independence
3. Improved ability to carry out some ADL
4. Completes ADLs more quickly
5. Needs less reminding during an ADL
6. Improved social interaction
7. Less depression
8. Less anxiety
9. Less frustration
10. Less anger
11. Better coping ability
12. Improved memory for some things
13. More social engagement
14. An overall better quality of life
15. More enjoyment of life
16. More better moments
17. Expresses appreciation more
18. More cooperative
19. How satisfied are you with your progress in this program?

3 = Completely. 2 = Somewhat. 3 = Not at all

1. Overall do you feel you are:

5 = Much better. 4 = Better. 3 = About the same. 2 = Worse. 1 = Much worse

1. Would you recommend the MHS to your colleagues? 2 = Yes. 1 = No
2. Assent Form
   1. Assent Form for Minors with Down Syndrome

This handout is about the MapHabit Down Syndrome Study. The MapHabit System features an app for your smart phone device or tablet that seeks to help you learn and gain independence.

We are asking that you join our study because we want to learn more about the health of children with Down Syndrome

You will be a part of a study that includes other children with Down Syndrome at different locations across the United States!

A group of professionals called an IRB reviewed this study for us. Their job is to assure that this study is safe and that participants like you are protected.

You will be using the MapHabit System and application for 6 months. You may have a few visits to a study site OR a virtual study meeting during the time you are in the study. You will come with your study partner. Your study partner will be a parent, other family member, or caregiver!

Your study partner will complete a set of questionnaires on your behalf at the start and at the end of the study. You are only expected to use the MapHabit System daily with the supervision of your study partner.

If you have any questions, you and your study partner may ask a member of the study team!

This study is private. Only your study partner and the study team will know that you are participating in this study. Your personal information (name, address, pictures, audio, or video) will not be shared beyond your designated study partner and the study team.

We anticipate that using the MapHabit System will be a positive and fun learning opportunity however—you do not have to be in this study if you do not want to and there are no consequences if you choose not to participate. You will always have the right to stop at any time.

Thank you for learning about the study and we hope that you join us! If you have any questions, you and your study partner may ask the study coordinator or a member of the study team!

18. References

[1] Jack CR Jr, Petersen RC, Xu Y, O'Brien PC, Smith GE, Ivnik RJ, Tangalos EG, Kokmen E. *Rate of medial temporal lobe atrophy in typical aging and Alzheimer's disease.* Neurology. 1998 Oct;51(4):993-9.

[2] Serrano-Pozo A, Frosch MP, Masliah E, Hyman BT. *Neuropathological alterations in Alzheimer disease.* Cold Spring Harb Perspect Med. 2011 Sep;1(1):a006189

[3] Farrand P, Hussain F, Hennessy E. *The efficacy of the 'mind map' study technique.* Medical Educ. 2002; 36:426-431.

[4] Mento AJ, Martinelli P, Jones RM. *Mind mapping in executive education: applications and outcomes*. J Manage Develop. 1999;18(4).

[5] Jeste DV, Palmer BW, Appelbaum PS, et al. A New Brief Instrument for Assessing Decisional Capacity for Clinical Research. *Arch Gen Psychiatry.* 2007;64(8):966–974. doi:10.1001/archpsyc.64.8.966

[6] Hartley, D., Blumenthal, T., Carrillo, M., DiPaolo, G., Esralew, L., Gardiner, K., Granholm, A. C., Iqbal, K., Krams, M., Lemere, C., Lott, I., Mobley, W., Ness, S., Nixon, R., Potter, H., Reeves, R., Sabbagh, M., Silverman, W., Tycko, B., Whitten, M., … Wisniewski, T. (2015). Down syndrome and Alzheimer's disease: Common pathways, common goals. *Alzheimer's & dementia: the journal of the Alzheimer's Association*, *11*(6), 700–709. <https://doi.org/10.1016/j.jalz.2014.10.007>

[7] Down syndrome. (2018, March 08). Retrieved June 10, 2020, from https://www.mayoclinic.org/diseases-conditions/down-syndrome/symptoms-causes/syc-20355977

[8] Mahoney, G., Perales, F., Wiggers, B., &amp; Herman, B. B. (2006). Responsive Teaching: Early intervention for children with Down syndrome and other disabilities. Down Syndrome Research and Practice, 11(1), 18-28. doi:10.3104/perspectives.311
