# Supplementary material for: A feasibility study demonstrating that independence, quality of life, and adaptive behavioral skills can improve in children with Down syndrome after using assistive technology
Source: PLoS One. 2023 May 24;18(5):e0284738. doi: 10.1371/journal.pone.0284738 (PMC10208506; doi:10.1371/journal.pone.0284738)
Supplement: Attachment — Submitted filename: Response to Reviewers.docx [file pone.0284738.s003.docx]

Dear Dr. Verduci,

Thank you for giving us the opportunity to submit a revised draft of our manuscript previously titled “Independence, Quality of Life, and Adaptive Behavioral Skills Improved in Children with Down Syndrome After Using Assistive Technology” to *PLOS ONE*, now titled “A Feasibility Study Demonstrating that Independence, Quality of Life, and Adaptive Behavioral Skills Can Improve in Children with Down Syndrome After Using Assistive Technology” (see Reviewer Comment 1). We are grateful for the time and effort that you and the reviewer have dedicated in providing valuable feedback on our manuscript. We appreciate the insightful comments from you and the reviewer. We were able to incorporate changes to reflect the suggestions that were provided. Below are the point-by-point responses to the editor and reviewer’s comments and concerns:

**Comments from Editor**

**Comment** **1**: *Please ensure that your manuscript meets PLOS ONE's style requirements, including those for file naming*

**Response**: Thank you for this reminder. We have reviewed the manuscript and made changes that meet PLOS ONE’s style requirements.

**Comment** **2**: *Please provide additional details regarding participant consent. In the ethics statement in the Methods and online submission information, please ensure that you have specified what type you obtained (for instance, written or verbal, and if verbal, how it was documented and witnessed). If your study included minors, state whether you obtained consent from parents or guardians. If the need for consent was waived by the ethics committee, please include this information.*

**Response**: We have added the details mentioned in the comment above into the Methods section of the revised manuscript.

**Comment** **3**: *We note that you have provided funding information that is not currently declared in your Funding Statement. However, funding information should not appear in the Acknowledgments section or other areas of your manuscript. We will only publish funding information present in the Funding Statement section of the online submission form…* *Please remove any funding-related text from the manuscript and let us know how you would like to update your Funding Statement…* *Please include your amended statements within your cover letter; we will change the online submission form on your behalf.*

**Response**: We have removed the funding information from the Acknowledgements section and included our amended statements within our new cover letter.

**Comment** **4**: *Please confirm that this does not alter your adherence to all PLOS ONE policies on sharing data and materials, by including the following statement: ""This does not alter our adherence to PLOS ONE policies on sharing data and materials.” (as detailed online in our guide for authors http://journals.plos.org/plosone/s/competing-interests). If there are restrictions on sharing of data and/or materials, please state these. Please note that we cannot proceed with consideration of your article until this information has been declared. Please include your updated Competing Interests statement in your cover letter; we will change the online submission form on your behalf.*

**Response**: We confirm this does not alter our adherence to PLOS ONE policies on sharing data and materials. The updated Competing interests statement is included in our amended statements within our new cover letter.

**Comment** **5**: *We note that you have stated that you will provide repository information for your data at acceptance. Should your manuscript be accepted for publication, we will hold it until you provide the relevant accession numbers or DOIs necessary to access your data. If you wish to make changes to your Data Availability statement, please describe these changes in your cover letter and we will update your Data Availability statement to reflect the information you provide.*

**Response**: We confirm the data availability statement upon acceptance.

**Comment** **6**: *Please include your full ethics statement in the ‘Methods’ section of your manuscript file. In your statement, please include the full name of the IRB or ethics committee who approved or waived your study, as well as whether or not you obtained informed written or verbal consent. If consent was waived for your study, please include this information in your statement as well.*

**Response**: The full ethics statement has been included in the ‘Methods’ section of our manuscript file.

**Comments from Reviewer**

**Comment** **1**: *If it is a feasibility type of study, the word feasibility is to be added to the title.*

**Response**: The title has been revised to address this comment.

**Comment** **2**: *Data analyses section is to be placed in the method section and not in the results section.*

**Response**: The suggested change has been made in the manuscript.

**Comment** **3**: *Line 115, the publisher’s name for the statistical software and its version to be stated. The level of statistical significance acceptance is to be stated.*

**Response**: The suggested additions have been made in the manuscript.

**Comment** **4**: *Line 116-117, the use of the statistical tests for what comparison in the context of this study and the name of the correlation test is to be clearly stated.*

**Response**: The suggested additions have been made in the manuscript.

**Comment** **5**: *Effect size could be employed to determine the effect of the intervention.*

**Response**: See Response to Comment 6, below.

**Comment** **6**: *For a feasibility study, significance testing is not encouraged unless the sample size is properly/adequately powered (Line 166, 'limited sample size')*

**Response**: We have added a section to the discussion indicating that our study was underpowered for some analyses (e,g., further stratification of subject groups, effect size). Nevertheless, as we have indicated, the findings evidenced important behavioral outcomes to be further explored, as well as the need for researchers to be prepared to devote considerable resources to recruitment efforts and participant retention.

**Comment** 7: *The percentage figures are to be presented with at least one decimal point.*

**Response**: The suggested change has been made in the manuscript.

**Comment** 8: *Line 117-119, Line 120-125, the data to be presented in table form.*

**Response**: The suggested change has been made in the manuscript.

**Comment** 9: *Figure 2A could be presented in table form with median and IQR values rather than using a bar chart.*

**Response**: The suggested change has been made in the manuscript.

**Comment** **1**0: *Line 122-123, the flow of the sentence requires improvement.*

**Response**: The suggested clarification has been made in the manuscript.

**Comment** **11**: *Line 124, ‘two subgroups participants’ to be revised. To state ‘there was no statistical difference between the 18 and 8 participants.'*

**Response**: The suggested revision has been made in the manuscript.

**Comment** **12**: *Line 127, the name of the statistical test to be denoted in the Figure 2b footnote and in the data analyses section.*

**Response**: The suggested additions has been made in the manuscript and figure.

**Comment** **13**: *Figure 2a, statistical test to be denoted in the figure footnote. P value could be displayed on the figure rather than mentioned in the title.*

**Response**: The suggested additions and changes have been made in the manuscript and figure.

**Comment** **14**: *Figure 2b, *, *** and dark line to be denoted in the Figure footnote.*

**Response**: The suggested additions have been made in the figure.

**Comment** **15**: *CONSORT diagram to be revised and tweaked based on this study e.g., method, flow etc.*

**Response**: We request that the CONSORT diagram that was previously submitted be removed. In PLOS ONE’s [submission guidelines](https://journals.plos.org/plosone/s/submission-guidelines), the following statement is included: “*Clinical trials must be reported according to the relevant reporting guidelines, i.e. CONSORT for randomized controlled trials, TREND for non-randomized trials, and other specialized guidelines as appropriate*.” As our study is non-randomized, we believe the TREND statement checklist is appropriate. We have included a revised TREND statement in our resubmission.

**Comment** **16**: *Not all references comply with the journal format.*

**Response**: We have adjusted the references to comply with the journal format.

We look forward to hearing from you in due time regarding our submission and to respond to any further questions and comments you may have.

Sincerely,

Samuel S. Han

Clinical Lead

MapHabit, Inc., Atlanta, GA 30308

shan@maphabit.com

Tel: 207-991-1955
