# Supplementary material for: A feasibility study demonstrating that independence, quality of life, and adaptive behavioral skills can improve in children with Down syndrome after using assistive technology
Source: PLoS One. 2023 May 24;18(5):e0284738. doi: 10.1371/journal.pone.0284738 (PMC10208506; doi:10.1371/journal.pone.0284738)
Supplement: Attachment — Submitted filename: Response to Reviewers.docx [file pone.0284738.s004.docx]

Dear Mr. Cowley,

Thank you for giving us the opportunity to submit a revised manuscript titled “A feasibility study demonstrating that independence, quality of life, and adaptive behavioral skills can improve in children with Down syndrome after using assistive technology” to *PLOS ONE*. We are grateful for the additional time and effort that you and the reviewers have dedicated in providing valuable feedback on our initial revised manuscript. Below are the point-by-point responses to the reviewers’ comments and concerns:

**Comments from Reviewer #2**

**Comment** **1**: “*Abstract: The term ’nationally’ implies a reader knows where the study is conducted. Consider naming the country instead.”*

**Response**: We agree with the reviewer that ‘nationally’ is vague for an international audience. We have replaced all mentions of the word to the specific country: United States of America.

**Comment** **2**: “*Personally, I find it difficult to use the framing ‘the first study on X’”*

**Response**: We understand the reviewer’s caution with framing a study as the first to investigate on a particular subject. We have reframed the study as contributing to the expansion of research in an area that is still relatively new.

**Comment** **3**: *“I think the term significant should not be stated in the question”*

**Response**: Following the reviewer’s suggestion, we have removed the word ‘significant’ to reserve its use for discussing statistical outcomes.

**Comment** **4**: “*Introduction: I think the outline lacks a clear description of what is meant by feasibility of AT…Additionally, there is little to no description of what kind of AT that is the focus of the study, and why you choose to study the MapHabit System”*

**Response**: We have included additional details that define feasibility for the scope of our study and provided further rationale for using the MHS for the intervention.

**Comment** **5**: “*I would have preferred describing and provide the rationale for each construct/the feasibility take in the Introduction section”*

**Response**: To clarify the structure of the manuscript, we have made changes in the Introduction. In addition, we included new subheadings to the Method section, as suggested by the reviewer in the next comment.

**Comment** **6**: “*Method section. General: the Enrollment, consent, & clinical trial registration section would be a lot easier to read if there were subheadings.”*

**Response**: We have added subheadings to this section for better organization.

**Comment** **7**: “*L54: Name the country.”*

**Response**: The country (USA) is now named in the manuscript. It is now located in line 78.

**Comment 8**: “*L115…I don’t think the design allow for them being their own controls just because you measure pre- and post-intervention…Consider removing that argument or provide a clearer description, number of measurement points etc.”*

**Response**: We appreciate the reviewer’s comment. The sentences were removed and replaced to be more straightforward: “permitted us to obtain baseline measures and compare them with the post-intervention outcomes”.

**Comment** **9**: “*L130-135: Provide rational of why you used non-parametric tests…Also, in the results section you write: ‘one-sample Wilcoxon test’. Is this correct”*

**Response**:

We appreciate Reviewer #3’s discussion of the statistical aspects of the study. The Reviewer is correct, the test used for the analysis on line 136 should have been indicated as the Wilcoxon Signed Rank test, not the t-test. We have corrected the typo.

Additionally, Reviewer #3’s query about why nonparametric tests were used for the analyses (particularly for the QOL analysis) encouraged a second look. For the present study, the data sets were generated from relatively small samples (and possibly skewed data, because of the nature of the diagnosis of the participants). Accordingly, it was likely that our samples would yield scores from dependent measures that would depart from normality and potentially violate assumptions inherent to parametric tests (Seigel, 1956; Leech et al., 2002). Additionally, small sample sizes, as in the present study, will not cause the results derived from nonparametric analyses to be misleading to the extent that small sample sizes and outliers unduly can affect parametric tests (Hollander and Wolfe, 1973; McSeeney and Katz, 1978). Taking these points together, we feel confident our use of nonparametric tests in this study, including the QOL analysis, is appropriate, and we thank the Reviewer for the stimulating query.

**Comment** **10**: “*Method and Results. You have not mentioned the attrition or analysed what that meant for your conclusions.”*

**Response**: We agree with the reviewer that the attrition of our participants is a limitation of the study. This may be explained by the study being too burdensome in the COVID-19 pandemic environment. We have added this point as a limitation in the Conclusion.

**Comment** **11**: “*I think a conflict-of-interest statement should be added to the manuscript.”*

**Response**: Per the guidelines of *PLOS ONE*, a conflict-of-interest statement has already been submitted to PLOS ONE separately.

**Comments from Reviewer #3**

**Comment** **12**: *This well written research paper focuses on the use of AT from children with DS - It is suggested authors to give more information about the evaluation of their intellectual disability; the results may differ according the severity.*

**Response**: We believe that the reviewer’s point is a very important one, and further reinforces the importance of this feasibility study. Further studies that are more systematic, such as randomized controlled trials, will allow for more conclusive findings and the ability to stratify the participants by intellectual or developmental disability severity. The present study did not have sufficient power or number of participants. We have added this point as a limitation to our conclusions.

Sincerely,

Samuel S. Han

Clinical Lead

MapHabit, Inc., Atlanta, GA 30308

shan@maphabit.com

Tel: 207-991-1955

**References**

Hollander, M., & Wolfe, D. A. (1973). Nonparametric Statistical Methods (*Wiley Series In Probability And Mathematical Statistics).* Wiley.

Leech, N. L., & Onwuegbuzie, A. J. (2002). A Call for Greater Use of Nonparametric Statistics.

McSweeney, M., & Katz, B. M. (1978). Nonparametric statistics: Use and nonuse. Perceptual and Motor Skills, 46(3_suppl), 1023-1032.

Messick, S. (1957). Nonparametric Statistics for the Behavioral Sciences. Sidney Siegel. *McGraw-Hill*, New York, 1956. 312 pp. $6.50. Science, 126(3267), 266-266.
