# Supplementary material for: A feasibility study demonstrating that independence, quality of life, and adaptive behavioral skills can improve in children with Down syndrome after using assistive technology
Source: PLoS One. 2023 May 24;18(5):e0284738. doi: 10.1371/journal.pone.0284738 (PMC10208506; doi:10.1371/journal.pone.0284738)
Supplement: Attachment — Submitted filename: Response to Reviewers.docx [file pone.0284738.s005.docx]

Dear Dr. Zimmerman,

Thank you for giving us the opportunity to submit a revised manuscript titled “A feasibility study demonstrating that independence, quality of life, and adaptive behavioral skills can improve in children with Down syndrome after using assistive technology” to *PLOS ONE*. We are grateful for the additional time and effort that you and the reviewers have dedicated in providing valuable feedback that will enhance the manuscript. Below are the point-by-point responses to the reviewers’ comments and concerns:

**Comments from Reviewer #1**

**Comment** **1**: *Line 154, Line 177 and Figure 2, the name for one-sample Wilcoxon test or one-sample Wilcoxon signed-rank test to be standardised or alternatively one-sample Wilcoxon test (also referred as one-sample Wilcoxon signed-rank test) to be indicated.*

**Response**: We thank the reviewer for noting the inconsistency of naming the statistical analysis. We have clarified this by following the reviewer’s suggestion: “one-sample Wilcoxon test (also referred to as one-sample Wilcoxon signed-rank test)”.

**Comments from Reviewer #2**

**Comment** **1.A**: *I cannot see how you have addressed some of the concerns I raised. Responses for comment 4 and 5 say you have added rationale and descriptions in the manuscript, which I cannot find.*

**Response**: We believe comment 4 and 5 from the previous review have been addressed in the last rendition of the manuscript, seen in the Introduction (line 38-40; 48-50; 53-54) and Methods (line 100-105; 109-115).

**Comment** **1.B**: *Also, the attrition is only mentioned as a limitation, but no one knows how these participants' scores would have altered the conclusions. Still, the abstract does not mention this uncertainty.*

**Response**: We appreciate the reviewer noting the potential limitation of the incompletions seen in participation. We have clarified this as a limitation that could have affected the outcomes that we reported. This has also been addressed in the abstract.

Sincerely,

Samuel S. Han

Clinical Lead

MapHabit, Inc., Atlanta, GA 30308

shan@maphabit.com

Tel: 207-991-1955
